# Longitudinal association between parental-to-child-Maltreatment and self-reported Generalized Anxiety Disorder symptoms in Pakistani Adolescents

**DOI:** 10.1186/s13034-021-00387-1

**Published:** 2021-07-14

**Authors:** Maryam Pyar Ali Lakhdir, Ghazal Peerwani, Salman Muhammad Soomar, Apsara Ali Nathwani, Salima Farooq, Naureen Akber Ali, Asif Khaliq, Muhammad Masood Kadir, Syed Iqbal Azam

**Affiliations:** 1 Department of Community Health Sciences, Aga Khan University, First Floor, Stadium Road, P.O Box 3500, Karachi, Pakistan; 2 Department of Pediatrics and Child Health, Aga Khan University, Karachi, Pakistan; 3 Department of School of Nursing and Midwifery, Aga Khan University, Karachi, Pakistan; 4 School of Public Health and Social Work, Queensland University of Technology, Brisbane, Australia

**Keywords:** Child maltreatment, Generalized anxiety disorder symptoms, Adolescents, Pakistan, Prospective cohort

## Abstract

**Background:**

Parent-to-child maltreatment is considered one of the risk factors for Generalized Anxiety Disorder (GAD) symptoms, but this hypothesis has not been adequately tested in Pakistani settings.

**Aim:**

This study aimed to examine the association between parent-to-child maltreatment and the risk of developing GAD symptoms among adolescents.

**Methods:**

The association of none to rare, occasionally, and frequently parent-to-child maltreatment with the incidence of GAD symptoms was investigated in a sample of 800 adolescents aged 11–17 years who were followed for two years. Parent-to-child maltreatment was assessed using ICAST-C (International child abuse screening tool). GAD Symptoms were determined by SCARED (Screen for children anxiety-related disorders). Cox Proportional Algorithm was used to estimate risk ratios.

**Results:**

Among children with both uneducated parents, frequently maltreatment was associated with 7.31 (2.20–24.04) times the risk of GAD symptoms compared to none to rare maltreatment. In contrast, the risk of GAD symptoms in frequently maltreated children was 5.58 times (1.40–21.97) than negligibly maltreated children with either educated parent.

**Conclusion:**

The frequency of parent-to-child maltreatment is significantly associated with an increased risk of developing GAD symptoms in which parental education plays a crucial role. Parents should be imparted with the awareness of the consequences of child maltreatment. In Pakistani settings the need to have this awareness is even more necessary due to the culturally acceptable disciplinary measures used by parents.

## Introduction

Mental health disorders constitute 16% of the global burden of disease and injury in adolescents [1]. Statistics show one out of every five adolescents start experiencing at least one form of mental illness each year [2]. Individuals in the adolescent phase are emotionally, physically, socially, and mentally vulnerable as it is a crucial phase due to the initiation of transition from childhood to adulthood. This vulnerability leads to the development of many mental illnesses that might persist into adulthood, ultimately affecting an individual's overall health-related quality of life [3]. The most common mental illnesses encountered by adolescents include anxiety disorders (mainly generalized anxiety disorder-GAD), depression, and eating disorders [4].

GAD, defined as "excessive anxiety and worry, occurring more days than not for at least six months, about a number of events or activities,". GAD is a type of anxiety disorder characterized by continuous worry and anxiety along with several other symptoms such as: restlessness, functional impairment, sleep disturbance, gastrointestinal symptoms, chronic headaches and muscular cramps [5]. GAD symptoms are relatively common in adolescents, with a lifetime prevalence of 3% globally. If the time period is restricted to 3 months, the prevalence increases to 5% worldwide [6].

Estimates of GAD based on the symptomatology may vary worldwide; the reported prevalence of GAD in adolescents in the USA lies between 3 and 9%, whereas, in the United Kingdom, it is 0.7% [7]. Regionally more than a few studies have depicted the burden of GAD-related symptoms in children and adolescents. A study conducted in Urban India reported 20.9% GAD-related symptoms in children and adolescents [8]. In contrast, Iran reported a 0.54–20.18% burden among adolescents [9]. This vast range of prevalence reported by the aforementioned studies might be due several contextual and research methods. For example, the different cultural settings, different predefined diagnostic thresholds, availability of various tools to assess GAD based on symptomatology. The under-reporting of symptoms due to fear and stigma is another consideration [10].

There were limited studies in Pakistani settings that exhibited a definite burden of GAD-related symptoms in the adolescent population. One study conducted in Lahore, Pakistan, indicated 52% of adolescents have at least one symptom of GAD, whereas approximately 8% of adolescents have more than 4 GAD persistent symptoms [11].

Previous literature has highlighted multiple risk factors of GAD symptoms in adolescents. The most common factors include parent-to-child conflicts, parent–child relationship, parental control, hormonal changes (that occur during adolescence), genetic predisposition, stress, family conflicts, communication gap with parents, parental education, traumatic life experiences, academic stresses, physical changes, socioeconomic burden, conflicts in friendships and interpersonal relationships, succumbing to parental pressure, cultural norms and religious obligations [11–14]. Moreover, age was also indicated as one of the prime risk factors leading to GAD symptoms; early adolescence was linked to a greater risk than late adolescence [15]. Among all these significant risk factors, traumatic childhood experience due to parent-to-child maltreatment of any sort, including physical, emotional, mental as well as neglect, was found to be one of the most reported risk factors leading to the occurrence of GAD symptoms [16]. For adults who experienced any variant of parent-to-child mistreatment, the burden is reported to be 30–40% [17]. Statistics reveal 4–16% of children are maltreated by parents every year and, one in ten is neglected or psychologically mistreated [18]. 13% is the reported prevalence of parent-to-child maltreatment in developing countries [19, 20]. According to a previous study, 43% of children in Pakistan self-reported parent-to-child maltreatment and, amongst these 57% were neglected, 49% were physically maltreated, and 50% suffered from emotional maltreatment [21]. Another study indicated 25.5% of physical abuse and 17.9% of emotional abuse by parents among Pakistani children [22]. Statistics on child maltreatment in the Pakistani population are alarming compared to global and developing countries' statistics. The difference in numbers can be accounted for by the use of strict measures by parents for disciplining and punishing a child. Moreover, the normalization of beating and physical means to control a child might also explain the contrast in Pakistani settings. As per a Pakistani study (n = 192,789), 74% of the parents of school-going children and adolescents didn't receive any formal education. They never went to school, hence are considered illiterate. Of the remaining 26% of parents, 52% fathers, 30% mothers, and 24% both parents received formal school education and were considered literate [23]. Low parental literacy rates in Pakistan might also explain parents' use of abusive activities due to a lack of awareness [24].

The development of anxiety disorders due to parent–child maltreatment can be understood from previous research detailing how continuous and frequent exposure to child maltreatment interrupts hypothalamic–pituitary–adrenal axis functioning and leads to neurobiochemical changes in the brain [25]. These changes include, an increase in thalamic grey matter volumes of the subcortical region of the brain which can result in the development of GAD symptoms [26, 27]. Moreover, parents' constant maltreatment can disrupt an adolescent's sense of safety resulting in various fears, apprehensions, cognitive deficits, and emotional vulnerability, leading to anxiety disorders [28]. GAD and related symptoms have major public health implications predominantly in adolescents. GAD is considered as a prodrome of chronic depression [29]. Moreover, GAD is linked with developing sleep disorders, eating disorders, and other psychological comorbidities [30]. The long-term effects of GAD symptoms personally, socially, and economically negatively influence a person wellbeing and quality of life. As depicted above, if the immediate diagnosis is not treated it can result in further comorbities creating further burden upon health care systems. Therefore, addressing GAD is a healthcare priority. The need is even more urgent within developing countries where stigma is a barrier to self reporting early symptoms of GAD. Furthermore, the escalating burden of anxiety disorders in adolescents, creates a need to identify predictors of GAD early so symptoms can be appropriately treated.

In Pakistani cultural settings where strict disciplinary measures are considered standard practice [21, 31], parent-to-child maltreatment is extremely prevalent. Parent-to-child maltreatment can lead to various anxiety disorders, including GAD. Adolescents constitute a large portion of the Pakistani population, and their psychosocial health must be adequately supported to prevent further detrimental consequences. There is currently a gap in research which neglects to appreciate the Pakistani context and how potential risk factors (namely parent-to-child maltreatment), can lead to anxiety disorders in adolescents.Furthermore, the definite burden of GAD symptoms and its association with parent-to-child maltreatment in adolescents has not been investigated within Pakistani conextx. Thus the aim of this study was to determine the incidence of GAD symptoms; and to determine the unified role of parental education and frequency of parent-to-child maltreatment onto risk of developing GAD symptoms along with other risk factors in Pakistani adolescents.

## Methodology

### Study design

A prospective cohort study was conducted to determine the occurrence of GAD symptoms in adolescents aged 11–17 years in community settings of Karachi, Pakistan. This study was conducted in continuation of a cross-sectional survey that aimed to determine the burden of parent-to-child maltreatment in 800 adolescents residing in 32 random clusters of Karachi, Pakistan, in 2015 [21, 31]. The parent-to-child maltreatment status of adolescents was post hoc categorized into three categories based on frequency, namely none to rare, occasional, and frequent child maltreatment. Adolescents recruited for the preliminary study were screened for the presence of existing psychiatric disorders; individuals with any such disorder were excluded from the primary study. Four psychological outcomes, including GAD symptoms, were assessed after two years, assuming that the living conditions, life events, and frequency of abuse from their parents remain the same as these were at the baseline in year 2015 [31] There was no change observed in their living conditions while visited for the follow-up to assess outcomes. The frequency of parent-to-child maltreatment was considered as a primary exposure for this cohort study. All adolescents under each category of primary exposure were followed for two years untill 2017 for the development of GAD symptoms.

### Study setting and sampling strategy

A multistage cluster sampling was adopted, and 32 urban and peri-urban areas (clusters) of Karachi, Pakistan, were systemically selected as the primary sampling unit. Karachi is a large city with seven districts and 18 towns. It is challenging to obtain a listing of each individual, but the list of 7750 blocks (cluster) of 125–250 households made by the Pakistan Bureau of Statistics (PBS) were available and thus used. The sampling frame was available for 15 towns comprising 80 random clusters, and each cluster had a minimum of 125 households. The study targeted one-third of all households per cluster, that is, 25 households per cluster. Out of 80 clusters, 32 clusters were randomly selected through systematic random sampling by calculating cluster size and clusters from each town, resulting in a diverse group from various cultures and socioeconomic status. Twenty-five (25) households were selected from each of these 32 clusters through systematic random sampling.

The selection of a household was based on the presence of at least one child within thehousehold. A total of 800 households (secondary sampling units) were systematically selected from the primary sampling unit. An eligible adolescent (tertiary sampling unit) was selected randomly and examined for GAD symptoms from each household. Only one child from each household was recruited in the study. Referring to the first sampled household within clusters, if the house was found to be locked, then that house was re-visited a maximum of two times on the same day. After several attempts the house was then excluded from the sample and replaced with another household to complete the sample size. The next step considered if the household included a child and if the child was eligible for the study. If there was a child among residents but was not present then, the household was revisited a maximum of two times the same day after that; the house was excluded from the sample. In the presence of two or more eligible children in a household, balloting (simple random sampling) was done to select one. After a follow-up of 2 years, the retention rate was 93.8% (n = 751) out of the total participants.

### Study population

All children, male and female, aged between 11–17 years that were a part of the preliminary study, were followed for 2 years. The eligibility criteria for inclusion of participants included: a child had to live at home with at least one biological parent and would continue residing in Karachi in that particular area for a minimum of the study period. Moreover, adolescents' ability to give oral assent and written informed consent was a requirementfor inclusion in the study. Children with known severe physical or mental disabilities or severe chronic illnesses and psychiatric disorders were excluded from the study.

The study staff consisted of a male and a female psychologist, who visited the households of all the eligible participants. The staff were trained in the informed consent process, conducting interviews, and completing the questionnaires.

After obtaining assent from the adolescents and informed consent from their mothers/guardians, a structured study tool was administered to all the participants. Boys were interviewed by a male interviewer while the females by female staff. The study tool consisted of sociodemographic information, SCARED scale, ICAST-C, parental and familial characteristics. Depending on the participant's preference, the tool was administered in either English or Urdu.

#### Follow-up sample

**Fig. 1:**
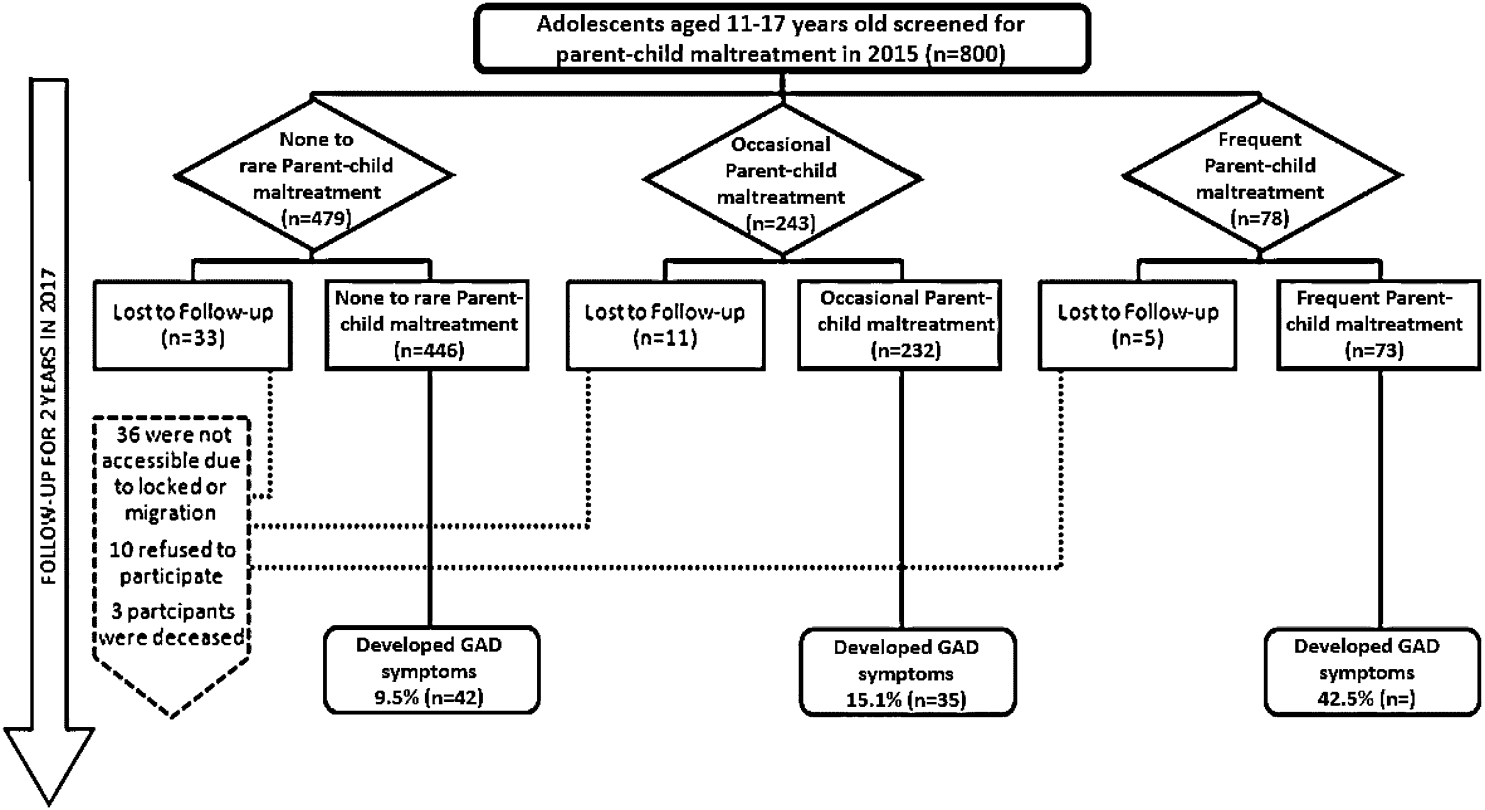
Flowchart of participants in the cohort study

The total sample size in the primary study consisted of 800 adolescents [31]. Final analysis of this study was carried out using 751 (93.8%) adolescents as 49 participants were lost to follow up. The reasons for loss to follow up along with the distribution of participants as per the status of primary exposure are elaborated in Fig. 1.

### Study variables

#### Dependent variable

GAD symptoms were measured with Screen for Child Anxiety Related Disorders (SCARED) scale [32]. SCARED isvalidated scale based on symptomatology, comprising of 5 domains with 41 items rated on a 3-points Likert scale starting from 0 to 2. point 0 indicates 'not truly and hardly even true,' 1 indicates 'somewhat truly or somewhat true' and two means 'very true or very often.' This instrument has been used previously in Pakistanisettings and has been field tested regionally [33]. One of the SCARED scale domains is based on symptoms of GAD, a score of 9 or more on item numbers 5, 7, 14, 21 23,28, 33, 35, and 37 indicates the presence of GAD symptoms, and it was considered as a binary variable. As per previous literature, the observer's reliability of this scale is appropriate for this study because the Cronbach alpha of 0.87 was reported [34].

#### Independent variables

##### Primary exposure

Parent-to-child maltreatment status in the primary study was assessed by the International child abuse screening for the child (ICAST-C) adapted from the International Society for the prevention of child abuse and neglect [35]. This is a validated self-reported instrument comprising 30 items rated on a 4 point Likert scale starting from 1 = never to 4 = many times. Three items in this scale had scores in descending order, whereas the rest were in ascending order. This instrument is considered reliable for assessing parent-to-child maltreatment by aprevious study with the Cronbach alpha of 0.72–0.855 [35]. As there was no definite cutoff for categorizing frequency of parent–child maltreatment, standard deviation (11.98) of mean ICAST-C score (51.71) was added and subtracted once to make categories of frequency of parent–child maltreatment. Participants having score of ≤ 39.73 were classified as having none to rare maltreatment (n = 479), score > 39.73 and < 63.69 was indicative of ocassional maltreatment (n = 243) and score ≥ 63.69 suggested frequent child maltreatment (n = 78). The primary exposure was categorized into three categories based on the frequency of maltreatment to the child as none to rare (n = 479), occasional (n = 243), and frequent maltreatment (n = 78). This tool covers physical victimization, psychological victimization, and neglect by parents tothe child. Sexual victimization from parents to the child was excluded from the primary study due to cultural restrictions.

#### Other covariates

Association of various covariates including age, gender, type of family system, presence of physical, verbal, and sibling abuse in the family, parental use of substance abuse, intergenerational abuse, history of parents psychiatric illness, history of maternal domestic violence, BMI, socioeconomic status, stress at home perceived by parents, parental employment and education status, child education status and birth order with the occurrence of GAD symptoms were assessed.

### Data collection and management methods

Face-to-face interviews were conducted by trained psychologists, using a structured questionnaire. The principal investigator and the field supervisor edited the filed questionnaire daily after the data collection. The revisions took place to ensure there wereno missing values, illogical or inappropriate entries, completeness, and accuracy of data. For missing values or inappropriate information principal investigator contacted the participant for correction and verification. The principal investigator did spot checks by administrating the questionnaire to the same participant to account for inaccuracies. Every effort was undertaken to ensure the completeness of data collected.

Moreover, before initiating data entry, another thorough check was completedto ensure the accuracy and completeness of data. Once the editing wascomplete, filled questionnaires were secured in a locked cabinet. All the participants were allotted unique ID numbers to ensure anonymity and confidentiality. Ethical approval was received from The Aga Khan University Ethical Review Committee (4816-CHS-ERC-17). To respect the autonomy of each participant, verbal assent and written informed consent was obtained.

### Data analysis

Data were entered in Microsoft Access (2007–2010) and analyzed using STATA version 15. As a y part of descriptive analysis, mean and standard deviation were reported for quantitative variables and frequency percentages for categorical variables. To determine the risk factors of GAD symptoms, cox proportional algorithm was used, and crude and adjusted risk ratios and 95% CI were reported using Cox after adjusting for multistage cluster random sampling technique.

## Results

A total of 800 adolescents were recruited in the preliminary study, out of which 49 (6.12%) were lost to follow-up. The number of participants who were lost to follow up from each level of exposure, along with reasons of loss to follow up, is detailed in Fig. 1.

The baseline characteristics of the participants as per the frequency of childhood maltreatment are displayed in Table 1. The mean age of children in this study was 13.13 (1.84) years. There were no differences in the mean ages among all levels of exposure. Among infrequent to frequent maltreated children, 144 (62%) and 47 (64%) were males, suggesting that males are more likely to endure parent-to-child maltreatment. Occasionally 123 (53%) and frequently maltreated children 46 (63%) were also more likely to be the middle child in the family.

**Table 1:** Baseline characteristics of key indicators of participants by frequency of parent-to-child maltreatment

Baseline characteristics	Total N = 751	None to rare parent-to-child maltreatment 446 (59%)	Occasional parent-to-child maltreatment 232 (31%)	Frequent parent-to-child maltreatment 73 (10%)
Age*	13.13 (1.84)*	13.29 (0.09)*	12.90 (0.11)*	12.84 (0.21)*
Gender				
Male	397 (52.86)	206 (46.19)	144 (62)	47 (64.38)
Female	354 (47.14)	240 (53.81)	88 (37.93)	26 (35.62)
Extended family system	325 (43.38)	184 (41.26)	111 (47.84)	30 (41.10)
Physical abuse within family	117 (15.58)	40 (8.97)	48 (20.69)	29 (39.73)
Verbal abuse/quarrel within family	425 (56.59)	206 (46.19)	157 (67.67)	62 (84.93)
Bullying and mistreating by siblings	376 (50)	162 (36.32)	152 (65.52)	62 (84.93)
Overweight/obese	195 (26)	137 (30.72)	44 (18.97)	14 (19.18)
Total Family members*	8.74 (4.84)*	8.23 (0.20)*	9.07 (0.32)*	10.78 (0.81)*
Parental use of substance abuse	246 (32.76)	126 (28.75)	88 (37.93)	32 (44)
Intergenerational abuse	367 (48.87)	242 (54.26)	94 (40.42)	31 (42.27)
History of parental psychiatric illness	192 (25.57)	103 (23.09)	63 (27.16)	26 (35.62)
Stress home environment perceived by parents	287 (38.22)	148 (33.18)	95 (40.95)	44 (60.27)
Not satisfied by family life	658 (87.62)	402 (90.13)	200 (86.21)	56 (76.71)
No familial support	655 (87.22)	391 (87.67)	203 (87.50)	61 (83.56)
Birth order				
First child	246 (32.76)	167 (37.44)	64 (27.59)	15 (20.55)
Middle child	342 (45.54)	173 (38.79)	123 (53.02)	46 (63)
Last child	163 (21.70)	106 (23.77)	45 (19.40)	12 (16.44)
Maternal History of domestic violence	244 (32.49)	118 (26.46)	93 (40.09)	33 (45.21)
Maternal employment status (housewife)	654 (87.32)	381 (85.81)	209 (90.09)	64 (87.67)
Paternal employment status (unemployed)	35 (5%)	17 (4.16)	12 (5.45)	6 (8.45)
Parental education status				
Both parents are educated	105 (13.98)	51 (11.43)	30 (12.93)	24 (32.88)
Either parent is uneducated	220 (29.29)	122 (27.35)	77 (33.19)	21 (28.77)
Both parents are not educated	426 (56.72)	273 (61.21)	125 (53.88)	28 (38.36)
Child education status (No formal education)	45 (5.99)	18 (4.04)	15 (6.47)	12 (16.44)
Socioeconomic status (composite wealth index)				
Low	100 (13.32)	55 (12.33)	31 (13.36)	14 (19.18)
Middle	542 (72.17)	310 (69.51)	179 (77.16)	53 (72.60)
High	109 (14.51)	81 (18.16)	22 (9.48)	6 (8.22)

^*^Means (Standard Deviation)

Specific familial characteristics were associated with severe childhood maltreatment. These characteristics include belonging to extended family (41%), presence of physical (39.73%), verbal (84.93%) and sibling abuse within the family (84.93), maternal history of domestic violence (45.21), parental use of substance abuse (44%) and stressful home environment (60.27%).

The occurrence of none to rare, occasional, and frequent parent-to-child maltreatment in the sample of 751 adolescents is 59% (n = 446), 31% (n = 232), and 10% (n = 73), respectively. The occurrence of GAD symptoms after two years of follow-up among frequently maltreated children is 42.5% (n = 31), whereas among occasionally maltreated and none to little-maltreated children, it is 15.1% (n = 35) and 9.5% (n = 42).

**Fig. 2:**
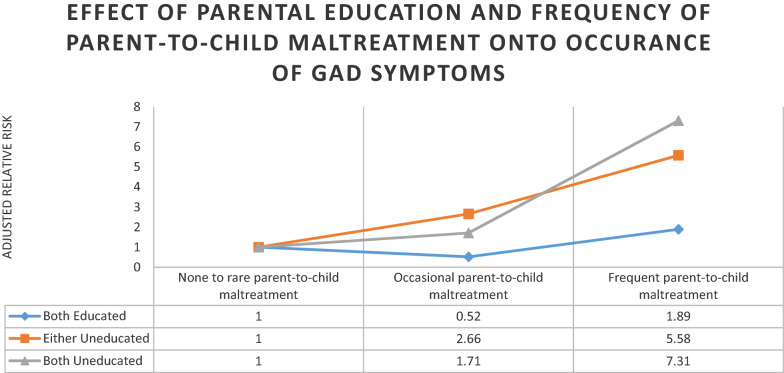
Effect of parental Education and frequency of parent-to-child maltreatment onto occurance of gad symptoms

Multivariable analysis using Multiple Cox proportional algorithm, the association between GAD symptoms with parent-to-child mistreatment and other predictors, is given in Table 2. The standardized risk of GAD symptoms was increased twofold in females compared to males (RR: 2.22 CI 1.56–3.15). Likewise, children with a maternal history of domestic violence had a 1.34 times greater risk of developing GAD symptoms than children with no history of maternal domestic violence (RR: 1.34 CI 0.98–1.83) adjusted for other variables. A significant interaction between parental education status and frequency of parent-to-child maltreatment suggests that in children with either uneducated parent, the risk of GAD symptoms was 5.58 times (CI 1.40–21.97) among frequently maltreated as compared to none to rare maltreated children. In comparison, for children whose mother and father had no formal education, exposure to frequent childhood maltreatment by parents was associated with a seven-fold increase in risk (CI 2.20–24.04) of developing GAD symptoms compared to none to rare exposure to parent-to-child maltreatment (Fig. 2).

**Table 2:** Adjusted risk ratio with 95% Confidence interval for covariates associated with GAD symptoms in adolescents in Karachi, Pakistan

Characteristics	Adjusted risk ratio	95% CI
Gender (female)	2.22	1.56–3.15
History of Maternal domestic violence	1.34	0.98–1.83
Parental education status and child maltreatment\		
Both parents with formal education		
None to rare parent-to-child maltreatment	–	–
Occasional parent-to-child maltreatment	0.52	0.16–1.61
Frequent parent-to-child maltreatment	1.89	0.89–4.31
Either parent with no formal education		
None to rare parent-to-child maltreatment	–	–
Occasional parent-to-child maltreatment	2.66	1.19–5.81
Frequent parent-to-child maltreatment	5.58	1.41–21.97
Both parents with no formal education		
None to rare parent-to-child maltreatment	–	–
Occasional parent-to-child maltreatment	1.71	1.09–2.63
Frequent parent-to-child maltreatment	7.31	2.20–24.04

## Discussion

Childhood maltreatment was recognized as one of the potential risk factors associated with GAD. This study has observed an increasing trend between the incidence of self-reported generalized anxiety disorder (GAD) symptoms and parent-to-child maltreatment. A total of 14.8% of adolescents developed GAD symptoms, among which severely maltreated adolescents had a comparatively higher risk than mild and moderately maltreated adolescents. Previous literature reported almost half of the incidence of GAD symptoms in maltreated adolescents compared to this study [16]. The probable reason for this difference could be different disciplinary measures adopted by parents within Pakistan. According to previous data, multiple variants of childhood maltreatment including physical abuse [36], emotional trauma [37], sibling victimization, and bullying [38] are associated with the development of GAD symptoms in children and adolescents. The results of the present study depicted similar associated predictors of GAD.

One of the findings of our study indicated that gender has a significant role in the occurrence of GAD symptoms. The risk of GAD symptoms was found to be increased in females as compared to males. Various studies showed agreement with this finding, indicating how anxiety disorders, especially GAD, are associated with a greater burden in females than males [39, 40]. The likely reason for this association could be a higher tendency of females to worry and ruminate [41]. Moreover, their genetic makeup and hormonal changes during ovulation and menstruation could be another consideration making females more vulnerable top GAD symptoms [41, 42]. In comparison, males tend to have a lesser tendency to develop GAD symptoms due to cultural norms that promote greater stoicism and less expressiveness which leads to less endorsement and acceptance of anxiety symptoms. Moreover, it is socially acceptable for females to express fear and anxiety but not for males who are expected to be brave [40].

It was evident from this study that the history of maternal domestic violence was another factor that played an essential role in the development of GAD symptoms in adolescents. This finding was consistent with previously research. Previous findings have demonstrated how interparental violence, specifically maternal violence, can increase children and adolescents' vulnerability to developing GAD symptoms [43, 44]. Void of a protective shield by parents and family that helps in neutralizing stresses of adolescents is affected by interparental violence, which might be why exposure to maternal violence is a significant predictor of GAD symptoms [44].

The results also revealed that GAD symptoms were higher among moderately or severely maltreated adolescents whose parents had no formal schooling. Certain studies have previously shown agreement with this interaction, suggesting that child abuse and GAD symptoms are associated with the level of parental education [24, 45]. It was also reported that parent-to-child maltreatment is common among adolescents with fathers with lower education levels or no education [24]. One plausible explanation for this interaction is that uneducated parents expect their children to do well for the family economically to raise the standard of living; hence they impose disciplinary measures and resort to physical and emotional abuse, leading to the development of GAD symptoms in adolescents [24]. Moreover, lack of adequate parenting skills, anger management, and adequate communication skills in uneducated parents might be the reason for practicing punitive disciplinary practices [31] that could lead to anxiety disorders in adolescents. Moreover, lack of adequate education results in a dearth of knowledge, skills and competence which are essential for understanding adolescents cognitive functioning as well as meeting their safety, developmental and wellbeing needswhich may contribute to parent-to-child maltreatment [46].

One of the significant strengths of this study is the sufficient sample size with negligible loss to follow-up. An adequate follow-up period of 2 years to ensure adequate time for the development of outcome and the rigor of a prospective cohort study design to establish temporality and causality are the most prominent strengths of this study. Moreover, a multicluster approach was used to target participants from diverse backgrounds. This can assist to ensure generalizability/ external validity of the study.The limitations of the study include: the subjectivity of the outcome and primary exposure. Moreover, few variables of this study did not have any definite definition and were conceptualized and assessed based on operational definitions. Certain potential risk factors and predictors of GAD based on symptoms reported by literature like socioeconomic status, intergenerational abuse, history of parents' psychiatric illness, etc., were insignificant in this study.

## Conclusion

The present study's findings suggest that the frequency of parent-to-child maltreatment is significantly associated with an increased risk of developing GAD symptoms. Parental education was also shown to play a crucial role in maltreatment practices. Intervention strategies should be designed to educate and enlighten uneducated and unaware parents regarding the consequences of parent-to-child maltreatment. This is particularly significant in Pakistani settings, as adopting discplianry action on a child is culturally acceptable. These cultural practices emphasize the need to develop and implement policies and rigorous measures to ensure such practices arereduced and stopped. The results of this study also indicated that girls are more vulnerable to developing GAD symptoms; hence adequate emotional support, timely treatment, and counseling should be provided to them to prevent long-term adverse consequences. In Pakistan, where the girls are considered responsibility bearers and have certain restrictions imposed on them, proactive identification and treatment of symptoms isneeded. Moreover, the findings of this study emphasize children who witness maternal domestic violence are more prone to develop symptoms of GAD. Hence a safe home environment should be ensured for the wellbeing and protection of children. The results also indicate that evaluation for the GAD symptoms should be a priority for adolescents with a history of moderate to severe maltreatment. Moreover, it also emphasizes the need for clinicians and mental health workers to be aware of the childhood maltreatment histories of potential patients presenting with GAD symptoms to be appropriately addressed and treated.

## Data Availability

The data that support the findings of this study are available on request from the corresponding author. The data are not publicly available due to information that could compromise the privacy of research participants.
